# Ethiopia’s quest to champion emergency care systems

**DOI:** 10.2471/BLT.19.240440

**Published:** 2019-09-01

**Authors:** Amir Aman Hagos, Tsion Firew, Alegnta Gebreyesus, Boureima H Sambo, Teri A Reynolds

**Affiliations:** a Federal Ministry of Health of Ethiopia, Addis Ababa, Ethiopia.; b Department of Emergency Medicine, Columbia University, 622 West 168th Street, Suite VC- 260, NY 10032, New York, United States of America.; c Climate and Other Determinants of Health Cluster, World Health Organization, Geneva, Switzerland.; d Department for Management of Noncommunicable Diseases, Disability, Violence and Injury Prevention, World Health Organization, Geneva, Switzerland.

Emergency care for all acutely ill and injured persons is an important component of a functioning health system and is needed for the implementation of universal health coverage (UHC). Estimates suggest that in low- and middle-income countries 54% (24 300 000/45 000 000) of deaths are due to conditions that could be treated with emergency care.^1^

In May 2019, the World Health Assembly passed a resolution on emergency care (WHA72.16).^2^ The resolution, proposed by the Governments of Ethiopia and Eswatini, calls for increased investment in emergency care systems and emphasizes the importance of fully integrating emergency care into ongoing UHC planning processes.^3^ The resolution provides an opportunity for coordinated action across the health system and for raising awareness of how emergency care is essential to a range of issues, including the health sector’s response to violence and injuries and the acute manifestations of infectious and noncommunicable diseases.

More than 30 Member States co-sponsored the resolution^4^ and Member States from the World Health Organization’s (WHO) African Region made a statement reflecting the support of all its constituent nations. The statement highlighted the need for well-organized, safe and high-quality emergency care to address population health needs in African countries.

Categories of actions for Member States listed in the resolution include: developing policies and governance mechanisms to ensure timely emergency care for all, with an emphasis on effective coordination of prehospital and emergency unit care across levels of the health system; providing emergency care training across all levels and cadres of health-care providers; implementing standardized data collection on emergency care use and outcomes; and conducting assessments of emergency care organization and delivery, using a range of WHO tools and standards.

WHO is tasked to enhance the Organization’s capacity across all levels to deliver technical support; raise global awareness of the significance of emergency care among international donors and partners, promote collaboration by convening networks; provide tools and standards to support Member State efforts to strengthen emergency care delivery; and strengthen the evidence base for the contribution of emergency care to the health system.

While this resolution will not solve all the problems in emergency care, we believe it will orient policy-makers to create strategies for sustainable funding, effective governance and universal access to safe, high-quality, needs-based emergency care. These strategies will aim to ensure emergency care for all people, regardless of sociocultural factors and the ability to pay before receiving services. We believe that high-quality emergency care can only be attained through collaborative efforts of multisectoral networks. For example, as part of the efforts to achieve the sustainable development goals, the Federal Ministry of Health, Ethiopia, is leading a seven-sector action plan, UHC Plus, to accomplish Ethiopia’s health sector transformation. Emergency care will be a key component of this plan and specific emergency care indicators have been included to ensure that this area is not neglected. As part of its efforts towards health for all, WHO is piloting a set of tools that help countries deliver high-quality emergency care services.^4^ The Ethiopian health ministry is collaborating with WHO to implement some of the tools based on action priorities identified in the country’s Emergency Care System Assessment. Furthermore, Ethiopia is leading the largest implementation of WHO’s *Basic Emergency Care: approach to the acutely ill and injured* course^5^ and the WHO’s International Registry for Trauma and Emergency Care.^6^ Through these efforts, the country is leveraging health systems strengthening and district transformation, both integral components of high-quality service needed to ensure impact on emergency care outcomes. Ethiopia’s revised national health policy recognizes the need for a coordinated emergency care system, and we look forward to other countries reporting progress on this resolution at subsequent World Health Assemblies.
